# A Case of Solitary Amyloid Lung Nodule Treated With Surgery

**DOI:** 10.7759/cureus.67013

**Published:** 2024-08-16

**Authors:** Prashanth Reddy Yella, Prachi P Jagani, Ravi P Jagani, Priya Elsa Skaria, Abhinav Chandra

**Affiliations:** 1 Internal Medicine/Hospital Medicine, Yuma Regional Medical Center, Yuma, USA; 2 Pre-Medical Sciences, Richmond Gabriel University, Kingstown, VCT; 3 Family Medicine, Yuma Regional Medical Center, Yuma, USA; 4 Hematopathology, Yuma Regional Medical Center, Yuma, USA; 5 Oncology, Yuma Regional Medical Center, Yuma, USA

**Keywords:** serum protein electrophoresis (spep), pulmonary nodular amyloidosis, plasma cell dyscrasia, solitary lung nodule, immunoglobulin light-chain amyloidosis

## Abstract

Primary or light-chain (AL) (lambda) amyloidosis is a rare systemic disorder that is characterized by the misfolding of autologous proteins and the extracellular deposition of abnormally folded proteins composed of immunoglobulin light chains, often caused by plasma cell dyscrasias.

We present a unique case of a 57-year-old female with multiple comorbidities, including extensive smoking history and chronic kidney disease, who was incidentally discovered to have a left upper lobe lung nodule on a chest X-ray prompted by complaints of shortness of breath. The patient underwent biopsy of the lung nodule, and by utilizing the gold standard diagnostic technique of a Congo red stain, positive test results confirmed the diagnosis of AL amyloidosis. However, additional investigations, including bone marrow and fat pad biopsies, were negative for plasma cell dyscrasias. The patient subsequently underwent a wedge resection of the nodule, and a follow-up positron emission tomography-computed tomography (PET-CT) scan showed only post-surgical changes in the left upper lobe of the lung without evidence of disease progression or systemic involvement.

Given the asymptomatic and multisystem symptomology of most cases, treatment options for AL amyloidosis are individualized. This case discusses pulmonary nodular AL amyloidosis and highlights the diagnostic and treatment options for this disorder.

## Introduction

Amyloidosis is a rare disease wherein abnormal protein fibrils, known as amyloids, accumulate in various organs and tissues throughout the body. This buildup can lead to organ dysfunction and ultimately death. The most common organs affected include the heart (75%), kidneys (65%), lungs (30%), liver (15%), soft tissues (15%), nervous system (10%), and gastrointestinal tract (5%) [1]. Currently, over 60 different heterogeneous amyloidogenic proteins have been identified, out of which approximately 30 are known to be associated with human disease [2]. Depending on factors such as the amount, type, and location of the protein buildup, amyloidosis can present in different forms: either systemic or localized. The types of amyloidosis include systemic AL (primary), systemic AA (secondary), systemic wild-type ATTR (senile), systemic hereditary ATTR (familial amyloid polyneuropathy), and localized AL [3]. AL amyloidosis is a plasma cell proliferative disorder and may be associated with an underlying monoclonal gammopathy of undetermined significance (MGUS), multiple myeloma, or another plasma cell dyscrasia [2]. Systemic AL amyloidosis, the most prevalent subtype, results from the deposition of protein derived from immunoglobulin light-chain fragments that arrange in beta-pleated sheets forming fibrils. The most impacted organs tend to be the heart and kidneys with a rare involvement of lungs [3].

This case emphasizes the importance of considering unusual presentations of amyloidosis and focuses on the need for a thorough diagnostic workup to enable early detection and treatment initiation. By sharing this case, we aim to educate clinicians about the diagnostic modalities and challenges associated with amyloidosis and the treatment options based on the location and type of amyloidosis.

## Case presentation

**Figure 1:**
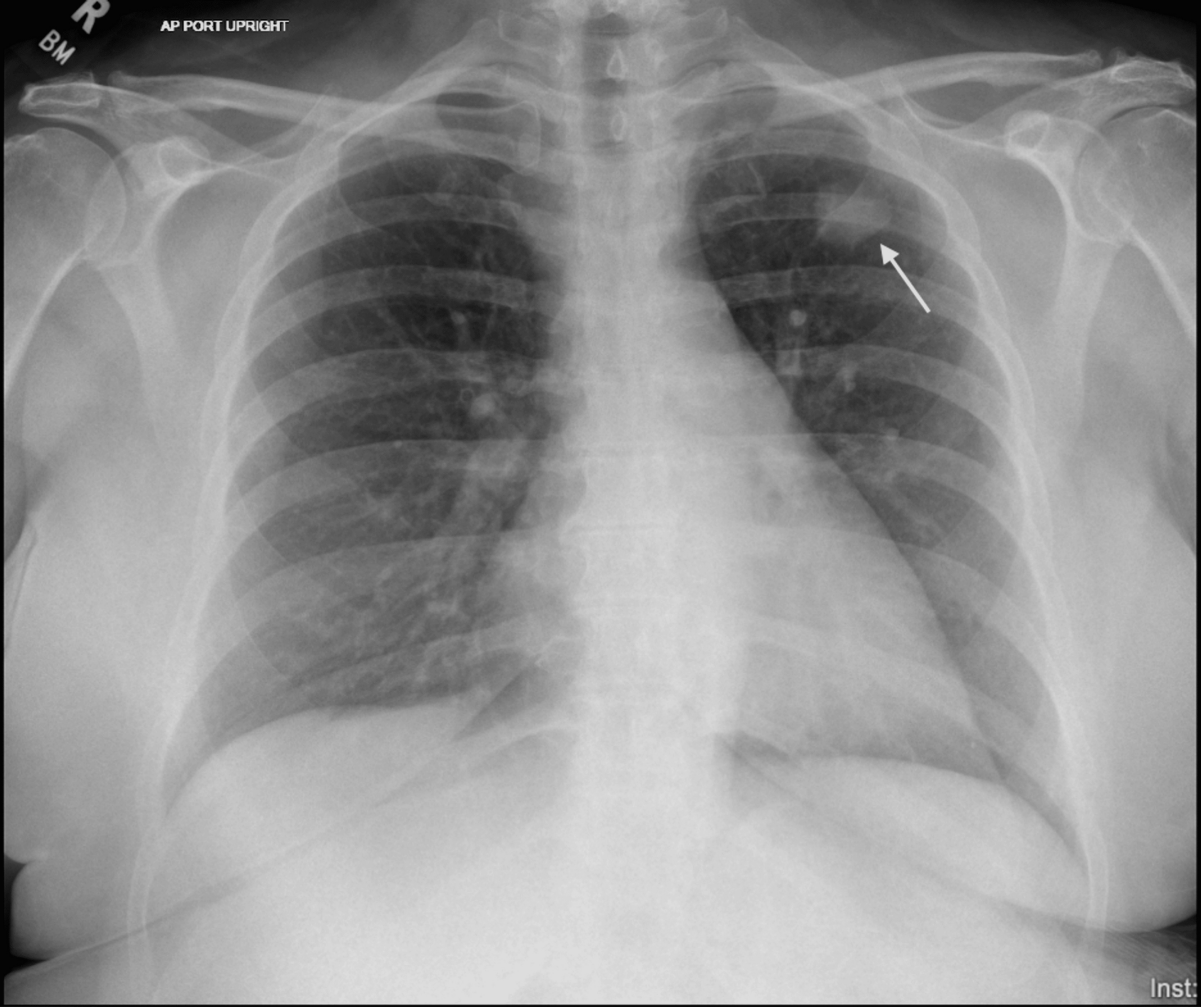
Chest X-ray showing a solitary lung nodule in the left lung (as highlighted by the arrow)

A 57-year-old female with a past medical history of chronic kidney disease stage 3, extensive smoking history (25 pack-years) quit two years ago, beta-thalassemia minor, hypothyroidism, hypertension, and seizure disorder presented to the pulmonology clinic after an incidental finding of a left lung nodule on chest X-ray (Figure 1) while undergoing the process of a colonoscopy.

**Figure 2:**
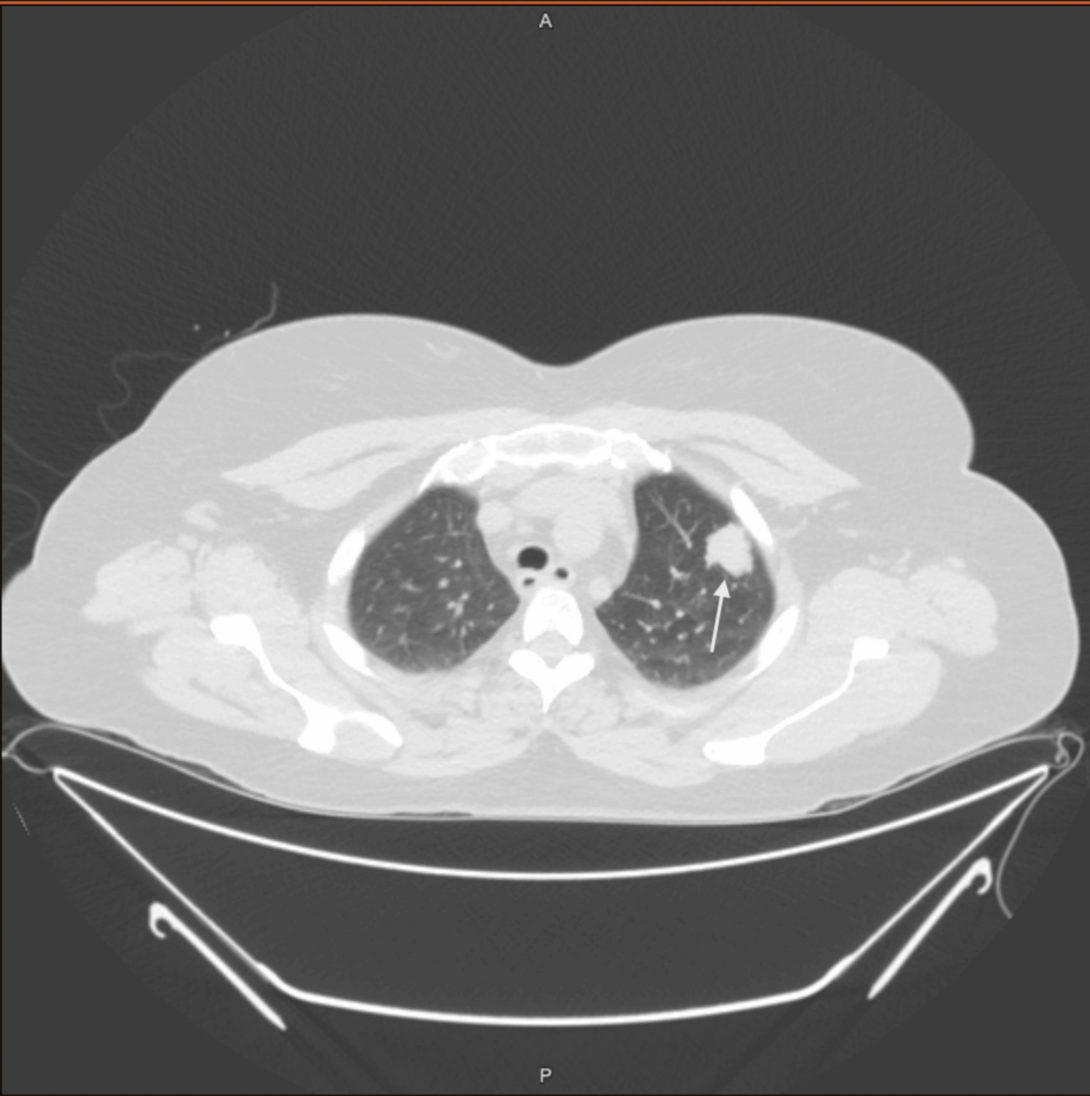
CT scan of the chest showing a 2×1.9 cm nodule in the upper lobe of the left lung (as highlighted by the arrow) CT: computed tomography

The chest X-ray was obtained as the patient had been complaining of intermittent shortness of breath, without any other associated symptoms. The chest X-ray was followed by a computed tomography (CT) scan of the chest without contrast which revealed a 2×1.9 cm nodule in the left upper lobe anteriorly (Figure 2).

**Figure 3:**
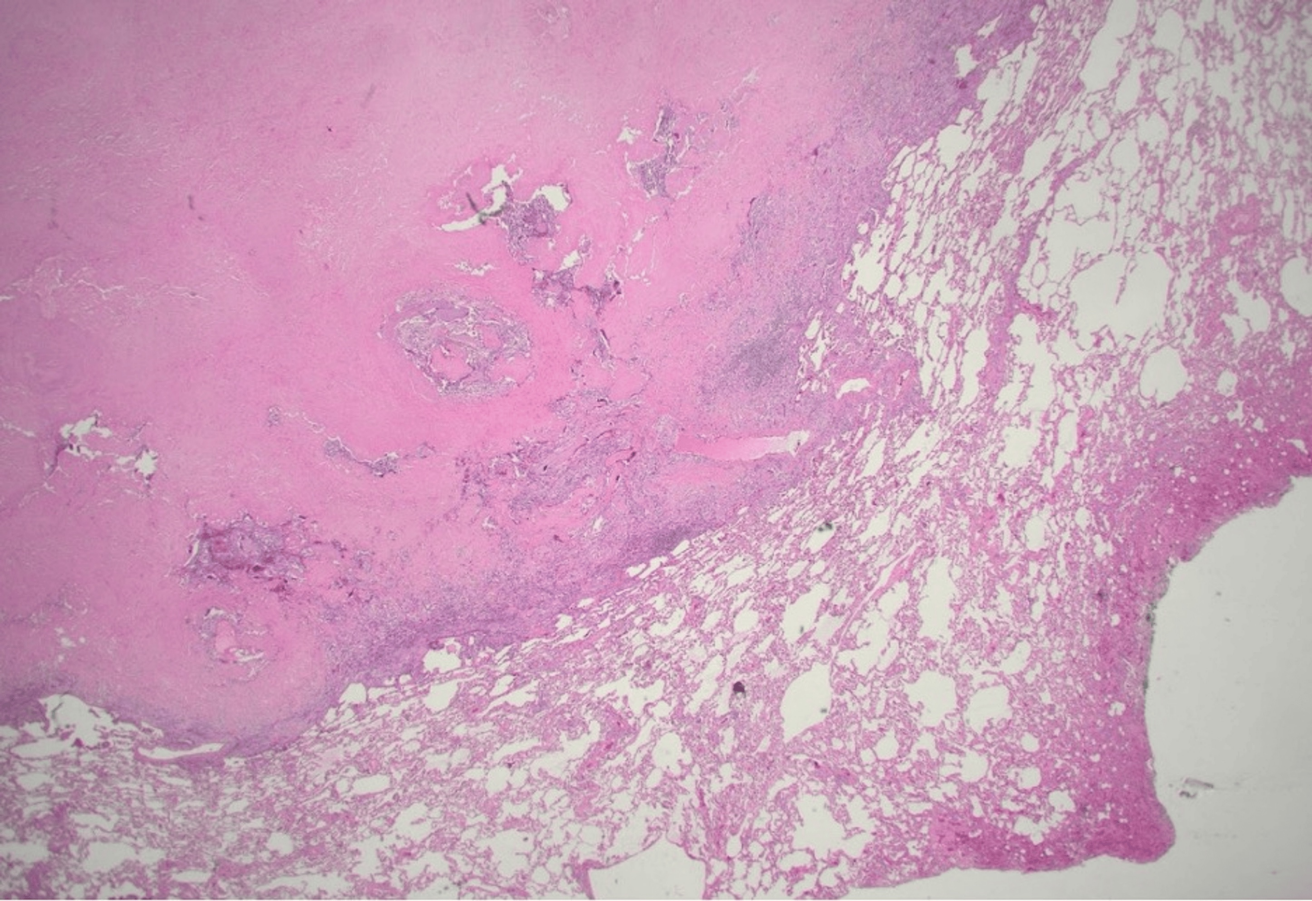
12.5× magnification, H&E stain: amyloid nodule in the peripheral lung parenchyma H&E stain: hematoxylin and eosin stain

**Figure 4:**
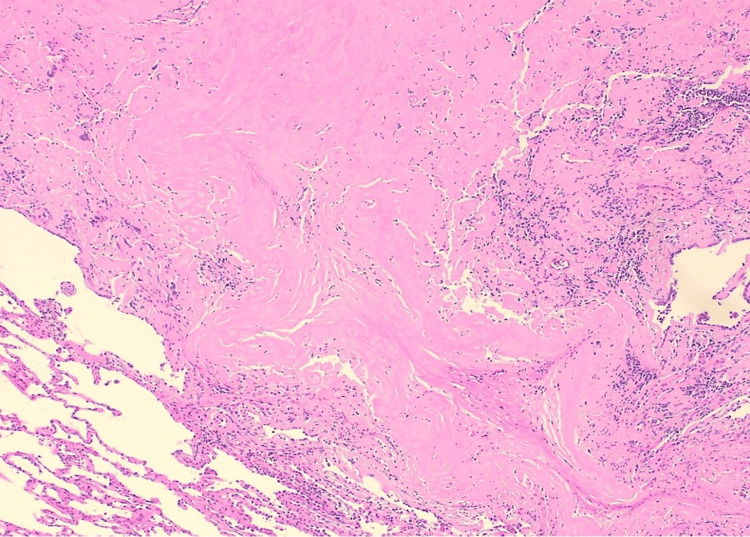
40× magnification, H&E stain: amyloid nodule in the lung parenchyma H&E stain: hematoxylin and eosin stain

**Figure 5:**
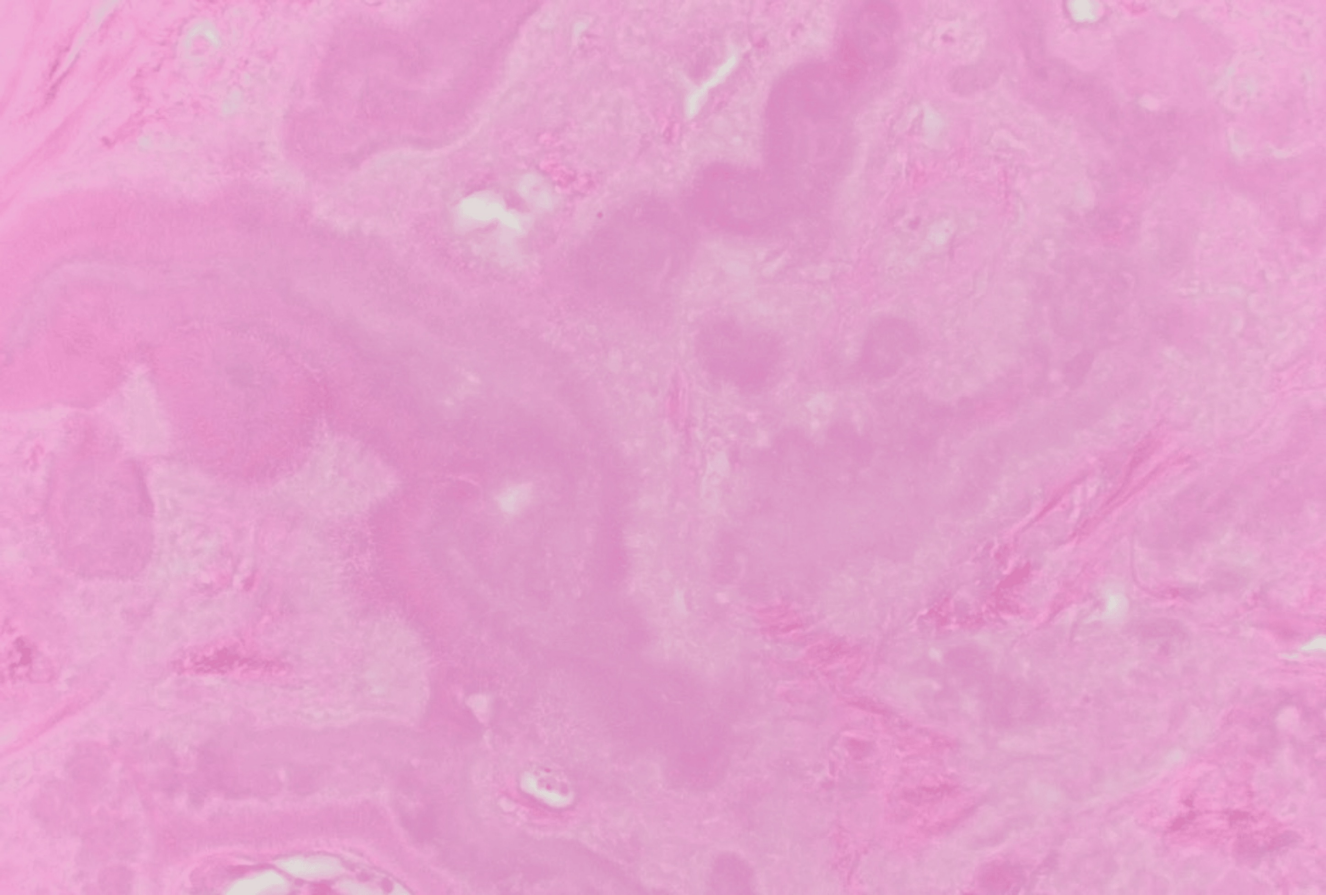
1000× oil immersion, H&E stain demonstrating amorphous eosinophilic extracellular amyloid deposits H&E stain: hematoxylin and eosin stain

**Figure 6:**
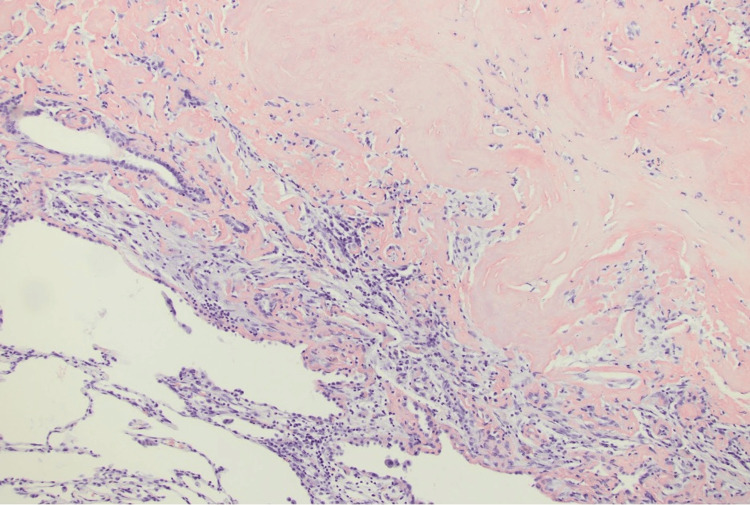
40× magnification: Congo red-positive amyloid in the lung parenchyma

**Figure 7:**
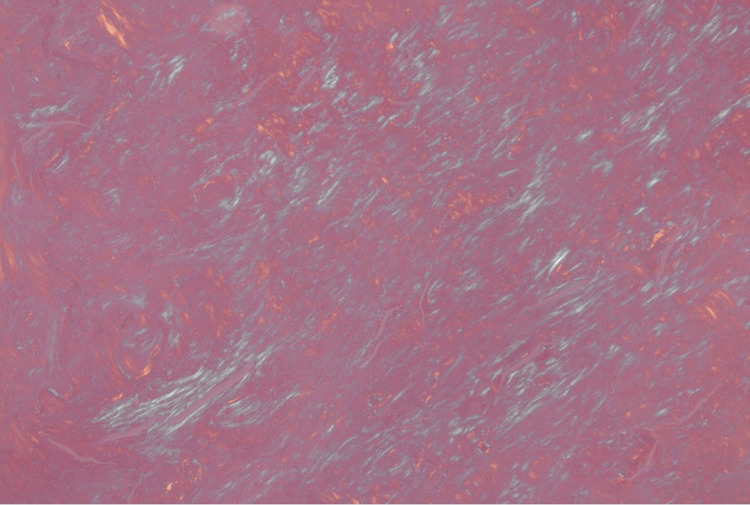
200× magnification: apple-green birefringence of amyloid under polarized light

Given the patient's extensive smoking history and new findings of the lung nodule, the patient underwent positron emission tomography (PET)-CT scan of the entire body which revealed a left upper lobe nodule with mild metabolic activity. The patient underwent a biopsy of the peripheral lung nodule which demonstrated an extracellular nodular deposition of amorphous and eosinophilic material (as shown in Figure 3, Figure 4, and Figure 5 with different magnification). It was positive for Congo red stain (as shown in Figure 6) and displayed an apple-green birefringence under polarized light (as shown in Figure 7).

Liquid chromatography with tandem mass spectrometry was performed on peptides extracted from Congo red-positive, microdissected areas of the paraffin-embedded specimen which confirmed ALH (lambda light chain, delta heavy chain) type. The overall histological findings were consistent with nodular pulmonary amyloidosis. Since AL amyloidosis is most commonly associated with plasma cell dyscrasias like MGUS and multiple myeloma, the patient underwent extensive workup for plasma cell dyscrasias including serum protein electrophoresis (SPEP), urine protein electrophoresis (UPEP), immunofixation, and serum free light chain assay (FLC). Initial laboratory studies showed a slightly elevated quantitative immunoglobulin A (IgA) level at 548 (range 61-356) and beta-2 microglobulin level at 3.67 (range 1.21-2.7). SPEP and UPEP were normal without any M-spike and not suggestive of plasma cell dyscrasias or Waldenström's macroglobulinemia. Serum FLC and immunofixation showed mildly elevated kappa free light chain level at 3.43 (0.33-1.94) and normal lambda free light chain level at 1.52 with mildly elevated free kappa-to-lambda ratio of 2.26 (0.26-1.65). Thereafter, the patient underwent bone marrow and fat pad biopsies, both of which were negative for involvement with plasma cell dyscrasia or amyloidosis. A 2D echocardiogram of the heart was noted to be normal, without any cardiac involvement with amyloidosis. As the patient was confirmed to only have a solitary lung nodule without any systemic involvement of amyloidosis, cardio-thoracic surgery was consulted, and she underwent wedge resection of the left lung nodule with a repeat pathology report showing negative margins. Biopsy was consistent with lung parenchyma with amyloid deposits with positive Congo red staining. A follow-up PET-CT scan showed only post-surgical changes in the left upper lobe of the lung without any new pulmonary parenchymal or pleural lesions. The scan no longer showed any metabolic changes as well. She is being followed regularly by the oncologist, and the follow-up labs and imaging including a CT scan of the chest continued to show no evidence of recurrence of amyloidosis.

## Discussion

Primary or light-chain (AL-type) lambda amyloidosis is the most prevalent form of systemic amyloidosis accounting for approximately 65% of the amyloidosis patients in the United Kingdom and 93% of those in China [4]. In the United States, the disease is extremely rare with an annual incidence of approximately 9-14 cases per million [5]. Typically, it presents at the median age of 64, with less than 5% of patients being under the age of 40. There is a slight male predominance with males accounting for 60% of patients; the gender ratio of males to females is 3:2 [3]. The underlying pathophysiology involves the deposition of plasma cell clones in the bone marrow that produce misfolded monoclonal immunoglobulin light chains, which form amyloid fibrils that circulate in the blood and accumulate in organs. This leads to organ dysfunction, most often in the heart and kidneys [6]. In our patient, who had a history of chronic kidney disease stage 3, the aspect of kidney dysfunction was already present. 

AL amyloidosis can occur as a localized or systemic variant and can present with a range of manifestations depending on the organs involved, including heavy proteinuria (in the nephrotic range), edema, hepatosplenomegaly, unexplained heart failure, impaired coagulation, and peripheral neuropathy [7]. These clinical features can occur alongside nonspecific symptoms like fatigue, alterations in taste, dry mouth, or unexplained weight loss, reflecting the systemic nature of the disease [7]. While pulmonary involvement is rare, it can manifest as three different types which are classified by location: nodular, diffuse alveolar-septal, and tracheobronchial [8]. Patients with nodular amyloidosis have a solitary nodule or multiple nodules on chest imaging and are usually asymptomatic. The diffuse alveolar-septal pattern of pulmonary amyloidosis is extremely rare and can present with dyspnea and nonspecific symptoms like weight loss and fatigue. Tracheobronchial amyloid infiltration can lead to hoarseness, stridor, airway obstruction, and dysphagia [9]. Initially, the symptoms tend to be nonspecific and may overlap with other common diseases. Due to the similarity in monoclonal plasma cell disorders, some patients diagnosed with AL amyloidosis may initially appear to have multiple myeloma or MGUS. It's worth noting that almost all cases of systemic AL amyloidosis are preceded by MGUS and 20% of MGUS patients develop multiple myeloma within 10 years [10]. Localized AL variant arises due to the local formation and deposition of AL amyloid fibrils within a tissue [11]. Treatment options differ depending on the type of pulmonary amyloidosis, systemic or localized variant, and its association with underlying plasma cell dyscrasias. 

Diagnostic criteria by the Mayo Clinic and International Myeloma Working Group require amyloid-related systemic syndrome (e.g., renal, liver, heart, gastrointestinal tract, or peripheral nerve involvement), positive Congo red stain or amyloid fibrils, evidence of light-chain relation, and evidence of monoclonal plasma cell disorder to validate a systemic AL amyloidosis diagnosis [12]. Of all patients with AL amyloidosis, approximately 2-3% will not meet the requirement for evidence of a monoclonal plasma cell disorder or amyloid-related systemic syndrome [12]. Among systemic syndromes, cardiac involvement is common (60-75%) and of utmost importance in determining the prognosis [2]. The gold standard diagnostic technique for AL amyloidosis is histopathological examination by tissue biopsy of the involved organ. Congo red and thioflavin S are the two major histological stains used to detect any form of amyloid as amyloid deposits exhibit an apple-green birefringent material under polarized light [13]. In patients with suspicion for systemic amyloidosis, SPEP and UPEP to detect monoclonal immunoglobulin chains, immunofixation to identify the type of light chain (kappa or lambda), and serum free light chain ratio analysis are done initially to detect evidence of monoclonal plasma cell disorder. If a monoclonal protein is present, then both abdominal fat pad aspirate and bone marrow biopsy are done. The fat pad biopsy, in which a fat sample is stained with Congo red dye, is a procedure to detect the characteristic feature of amyloid deposits in subcutaneous tissue [13]. Comparatively, a bone marrow biopsy is performed to evaluate plasma cell dyscrasia and the deposition of amyloid [14]. If AL amyloidosis is still suspected after negative fat pad aspirate and bone marrow biopsy, an organ biopsy may be necessary [14]. In our presentation, the patient initially complained of shortness of breath, leading to a chest X-ray that revealed a lung nodule in the left upper lobe. Through further imaging with a CT scan, the exact proportions of the lung nodule were discovered along with the exact location in the anterior left upper lobe. Furthermore, the patient underwent a biopsy of the lung nodule with Congo red staining which yielded positive results for AL amyloidosis. Thereafter, further evaluation was done to check for systemic involvement and underlying plasma cell dyscrasias, for which our patient underwent extensive diagnostic testing including SPEP, UPEP, immunofixation, and serum FLC along with bone marrow and fat pad biopsies, all of which were negative for plasma cell disorders or amyloid deposition. Additionally, an echocardiogram was performed which showed no evidence of cardiac involvement. Our patient already had a history of chronic kidney disease; however, due to lacking advanced-stage kidney involvement or a history of dialysis, amyloid deposits were not believed to have caused the kidney dysfunction. Overall, the patient did not exhibit any other obvious systemic symptoms of amyloidosis, and final histology findings were most consistent with a diagnosis of nodular pulmonary amyloidosis, a localized variant of AL amyloidosis, thus highlighting the varied clinical presentation of this condition. 

All patients diagnosed with AL amyloidosis must undergo a thorough evaluation to assess their eligibility for autologous hematopoietic cell transplantation. However, the majority of newly diagnosed patients, over 80%, are deemed ineligible for transplantation due to factors such as advanced age (<70 years), renal insufficiency, advanced heart failure, or multiorgan involvement [15]. In these cases, chemotherapy becomes the primary treatment approach, drawing similarities to the treatment strategies employed in multiple myeloma. Additionally, targeted medications, such as lenalidomide, may be prescribed to inhibit the growth of abnormal plasma cells and reduce amyloid protein production [16]. For fit patients who opt for disease-directed therapy, a four-drug combination of anti-plasma cell agents, including daratumumab, cyclophosphamide, bortezomib, and dexamethasone (Dara+CyBorD), has shown promising results in treating newly diagnosed AL amyloidosis, as demonstrated by the recently published phase III ANDROMEDA study [14]. In specific cases of pulmonary nodular amyloidosis, characterized by CT scan findings of a solitary nodule without any systemic involvement, the recommended treatment is resection followed by surveillance [3]. In our patient, an autologous hematopoietic cell transplant was ruled out due to the presence of a solitary lung nodule without systemic spread. Additionally, given the patient's chronic kidney disease, it is important to manage and optimize renal function through appropriate medical interventions. This is why after consultation from cardio-thoracic surgery, the patient underwent a wedge resection of the lung nodule, with subsequent pathology reports showing negative margins. A follow-up PET-CT scan demonstrated only post-surgical changes in the left upper lobe of the lung, with no new pulmonary parenchymal or pleural lesions. The scan also indicated the absence of any metabolic changes, suggesting a successful resection of the nodule. Our case is peculiar as we used no chemotherapy-based or disease-directed drugs, with the surgery being enough to remove the solitary nodule. 

The prognosis of AL amyloidosis varies significantly based on the nature and extent of organ involvement. When detected at an advanced stage, the long-term prognosis is generally poor, with a survival rate as short as four to six months due to cardiac or hepatic failure. However, patients with limited organ involvement can have a more favorable prognosis. With current therapy, these patients can expect a median survival of over five years [3]. In our patient, the overall survival rate is increased due to the successful resection of the lung nodule and minimal range of systemic symptoms. 

## Conclusions

This case of a solitary amyloid lung nodule treated with surgery underscores the complexity and varied presentation of AL amyloidosis. Despite being a rare condition, timely and accurate diagnosis is crucial for effective management and improving patient outcomes. The use of histopathological examination, particularly Congo red staining, remains the gold standard for confirming amyloidosis. Our patient's successful treatment through surgical resection without the need for chemotherapy highlights the importance of individualized treatment plans. The patient's follow-up PET-CT scans showing only post-surgical changes and no evidence of disease progression further underscore the potential for positive outcomes with appropriate surgical intervention. This case also emphasizes the necessity of a thorough diagnostic workup to rule out systemic involvement and plasma cell dyscrasias. In the future, heightened awareness and early intervention are key to managing this challenging condition, and further research is needed to refine treatment strategies and enhance long-term patient care.
